# Prognostic Factors of Oligometastasis After Stereotactic Body Radiotherapy: The Real-World Utility of the European Society for Radiotherapy and Oncology/European Organisation for Research and Treatment of Cancer Classification

**DOI:** 10.7759/cureus.60590

**Published:** 2024-05-19

**Authors:** Ryosuke Bessho, Haruka Uezono, Yosuke Ota, Shuichiro Miyazaki, Mitsuru Marudai, Hatamei Takabayashi, Kayoko Tsujino

**Affiliations:** 1 Department of Radiation Oncology, Hyogo Cancer Center, Akashi, JPN

**Keywords:** cancer prognosis, adverse effects, progression-free survival, stereotactic ablative body radiotherapy, oligometastases

## Abstract

Aim: The efficacy of local therapy for oligometastatic disease (OMD) remains unclear. This study aimed to evaluate the prognostic utility of the classification system for OMD and explore which groups may benefit from stereotactic body radiation therapy (SBRT).

Methods: This single-center retrospective study included 45 patients (52 sites) with solid tumors and 1-3 extracranial oligometastases who underwent SBRT for all metastases at our institution between January 2018 and December 2021. OMD states were classified based on the European Society for Radiotherapy and Oncology (ESTRO) and the European Organisation for Research and Treatment of Cancer (EORTC) classification system. Local control (LC), overall survival (OS), and progression-free survival (PFS) for each group were analyzed using the Kaplan-Meier method. Acute and late adverse events (AEs) were evaluated according to the Common Terminology Criteria for Adverse Events (CTCAE) version 5.0.

Results: The median follow-up period was 14 months (range: 0-48 months). The numbers of patients in the de novo (first diagnosis of OMD), repeat (previous history of OMD), and induced (previous history of polymetastatic disease) OMD groups were 15, 17, and 13, respectively. The LC rates at one year for the entire, de novo, repeat, and induced cohorts were 87.2%, 87.5%, 90.2%, and 83.9%, respectively (p=0.80). The one-year PFS rates for each group were 35.0%, 56.7%, and 29.9%, respectively (p=0.58). The one-year OS rates for each group were 80.0%, 86.2%, and 80.8%, respectively (p=0.50). Grade 2 or 3 AEs occurred in five patients (10.4%). No grade 4 or 5 AEs were observed.

Conclusions: SBRT is safe and highly effective for local control. Patients with repeat OMD demonstrated a trend of longer PFS, suggesting that this subgroup may benefit from local therapy at metastatic sites.

## Introduction

Oligometastatic disease (OMD) was proposed as a transitional state of limited metastatic spread between localized and widely disseminated disease [1]. Several clinical trials have demonstrated a prolonged progression-free survival (PFS) or overall survival (OS) by adding local therapy to the standard of care [2-4]. Other trials have shown that adding local ablation for oligometastatic disease prolongs therapy-free survival in patients receiving first-line systemic therapy [5] and undergoing surveillance [6]. However, OMD occurs in many clinical scenarios, and the specifications for OMD are different for each study. Based on the currently available evidence, oligometastatic disease eligible for curative-intent radiotherapy is defined as having 1-5 metastases, with a controlled primary tumor being optional, but all metastases can be safely treated [7]. However, the prognostic factors for OMD after local treatment are yet to be elucidated.

To standardize oligometastatic nomenclature and provide risk stratification, the European Society for Radiotherapy and Oncology (ESTRO) and the European Organisation for Research and Treatment of Cancer (EORTC) developed a comprehensive classification system and nomenclature for OMD [8]. This classification system is based on five disease characteristic factors. OMD is first differentiated into induced OMD (previous history of polymetastatic disease) and genuine OMD (no history of polymetastatic disease). Genuine OMD is differentiated into de novo OMD (first diagnosis of OMD) and repeat OMD (previous history of OMD). De novo OMD is classified into synchronous OMD (OMD diagnosed at less than six months after the diagnosis of primary cancer) and metachronous OMD (more than six months after the diagnosis of primary cancer).

Previous prospective clinical trials that investigated the efficacy of stereotactic body radiation therapy (SBRT) in patients with OMD have mainly focused on de novo OMD controlled by systemic therapy [9]. However, the efficacy of local treatment for repeat OMD and induced OMD has not been extensively evaluated [10].

In this study, we reviewed the treatment outcomes of SBRT in patients with OMD at our institution and identified which group of patients, according to the ESTRO/EORTC classification, may benefit from metastasis-directed therapy.

This article was previously presented as a meeting abstract at the 35th Annual Meeting of the Japanese Society for Radiation Oncology Number O18-1. Additionally, this article was previously posted to the Research Square preprint server on August 30, 2023.

## Materials and methods

We retrospectively evaluated patients who developed oligometastases and were treated with radiotherapy between January 2018 and December 2021 at the Hyogo Cancer Center. The eligibility criteria were patients aged ≥18 years and with Eastern Cooperative Oncology Group performance status (ECOG PS) of ≤2, patients with 1-3 extracranial metastases from non-hematologic malignancies, and those who underwent SBRT for all metastatic lesions. Intracranial metastases are typically documented separately from extracranial oligometastatic disease [7]. Therefore, patients with intracranial metastases were not included in our study.

The primary tumor was controlled in 97.8% (n=44) of the patients. One lung cancer patient with repeat OMD received SBRT for two distant metastatic lesions (the adrenal gland and bone) and the primary lesion because of a suspected local recurrence of the primary lesion. No apparent change in the size of the primary tumor nor relevant symptoms were observed, but the increased accumulation of fluorodeoxyglucose was detected by positron emission tomography/computed tomography (PET/CT), suggesting a local recurrence. The majority of patients were staged using PET/CT (n=35, 77.8%), while others were staged using computed tomography (CT) (n=10, 22.2%). All patients underwent CT simulations, employing four-dimensional CT for lung or liver tumors. Gross tumor volume was determined by visible lesions on CT scans, with the option to use MRI and/or PET/CT. Ultra-hypofractionated SBRT (range: 24-50 Gy, 2-5 fractions) was performed according to the Stereotactic Ablative Radiotherapy for the Comprehensive Treatment of Oligometastases (SABR-COMET) protocols [11] and other studies [12,13]. Other ultra-hypofractionated and moderate hypofractionated regimens were permitted by the discretion of the oncologists (i.e., the target was adjacent to the organs at risk). Dose prescriptions were mainly determined by the treatment sites and not modified by the OMD state. Systemic therapy was administered following SBRT in cases where a high risk of distant metastasis was suspected by the physicians. However, it was not implemented in cases where patients were not tolerable for systemic therapy or when effective systemic treatments were unavailable.

The treatment plan was generated in the Eclipse treatment planning system using the volumetric modulated arc method. The radiation therapy was performed by the Varian TrueBeam® linear accelerator (Varian Medical Systems, Inc., Palo Alto, CA) or Novalis TX® linear accelerator (Brainlab, Munich, Germany).

**Figure 1:**
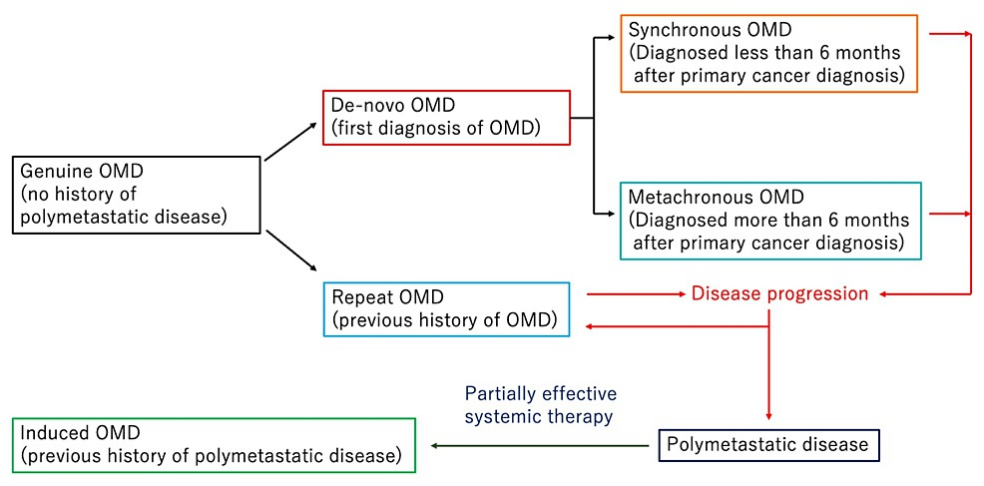
The classification of OMD and the transition of its state by ESTRO/EORTC OMD, oligometastatic disease; ESTRO/EORTC, European Society for Radiotherapy and Oncology/European Organisation for Research and Treatment of Cancer

The OMD status was classified according to the ESTRO/EORTC consensus recommendations (Figure 1), and its impact on local control (LC), PFS, and OS was evaluated. Furthermore, we analyzed various patient and treatment factors that might affect prognosis. Treatment-related toxicities were assessed according to the Common Terminology Criteria for Adverse Events (CTCAE) version 5.0. Adverse events (AEs) that occurred within three months after SBRT were considered acute, and AEs that occurred after three months were considered late AEs. Written informed consent was obtained from all patients for using their treatment-related data in retrospective studies. This study was conducted under the principles of the Declaration of Helsinki and was approved by the Institutional Review Board of the Hyogo Cancer Center (approval number: G-96).

Patients were typically followed up every 3-6 months after treatment. Thorough history taking and physical examination were performed in conjunction with the CT of the chest/abdomen/pelvis at every visit. PET/CT or magnetic resonance imaging was performed as needed.

The primary outcome of this study was PFS, which was defined from the time of SBRT to the time of either the enlargement of existing tumor volume, the development of new metastases, or death. The secondary outcomes of this study were overall survival (OS), defined as the time of SBRT to the time of death from any cause, and LC, defined as the time of SBRT to the enlargement of tumor volume in any irradiated lesion and treatment-related toxicity.

Clinical parameters were assessed between the three OMD groups using either Fisher’s exact test for categorical variables or the one-way analysis of variance (ANOVA) for continuous variables. A post hoc test was performed when either of these analyses indicated a significant difference. LC, OS, and PFS were analyzed using the Kaplan-Meier method. The log-rank test was used to calculate the statistical differences. Univariate and multivariate Cox regression models were used to assess the predictors of PFS. A p-value of less than 0.05 was considered to indicate a statistically significant difference. All statistical analyses were conducted using R software version 4.2.2 (R Foundation for Statistical Computing, Vienna, Austria).

## Results

The medical records of 45 eligible patients were reviewed. The median follow-up period after SBRT for the entire cohort and survivors was 14 months (range: 0-48 months) and 14 months (range: 3-48 months), respectively. The baseline patient characteristics are classified into three major categories per the ESTRO/EORTC classification, which are outlined in Table 1. The lungs were the most common primary site (20 patients, 44.4%), followed by the liver (eight patients, 17.8%), breast (four patients, 8.9%), bladder or ureter (three patients, 6.7%), and others (10 patients, 22.2%). The most frequently treated sites were the lungs (20 sites, 38.5%), bones (16 sites, 30.8%), and liver (10 sites, 19.2%). The majority of the patients (40 patients, 88.9%) treated had single oligometastatic lesions.

**Table 1:** Patient and tumor characteristics *Differences between groups were assessed using Fisher’s exact test for categorical variables and the one-way analysis of variance (ANOVA) for continuous variables **Others are composed of the rectum (n=2), kidney (n=2), stomach (n=2), thyroid (n=1), uterus (n=1), pancreas (n=1), and esophagus (n=1) ECOG PS, Eastern Cooperative Oncology Group performance status; OMD, oligometastatic disease; SBRT, stereotactic body radiation therapy; ESTRO/EORTC, European Society for Radiotherapy and Oncology/European Organisation for Research and Treatment of Cancer; BED, biologically effective dose

Variable						P-value*
ESTRO/EORTC classification		De novo	Repeat	Induced	Entire cohort	
Total (%)		15 (33.3)	17(37.8)	13 (28.9)	45 (100)	
Age (years)						0.218
	Median (range)	74 (52-92)	74 (56-85)	66 (51-84)	73 (51-92)	
Sex, N (%)						0.041
	Male	10 (22.2)	13 (28.9)	4 (8.9)	27 (60.0)	
	Female	5 (11.1)	4 (8.9)	9 (20.0)	18 (40.0)	
Primary tumor site, N (%)						0.074
	Lung	8 (17.8)	7 (15.6)	5 (11.1)	20 (44.4)	
	Liver	0 (0.0)	6 (13.3)	2 (4.4)	8 (17.8)	
	Breast	0 (0.0)	1 (2.2)	3 (6.7)	4 (8.9)	
	Bladder or ureter	1 (2.2)	2 (4.4)	0 (0.0)	3 (6.7)	
	Others**	6 (13.3)	1 (2.2)	3 (6.7)	10 (22.2)	
ECOG PS, N (%)						0.521
	0-1	14 (31.1)	14 (31.1)	10 (22.2)	38 (84.4)	
	2	1 (2.2)	3 (6.7)	3 (6.7)	7 (15.5)	
Treatment site, N (%)						0.060
	Lung	8 (15.4)	8 (15.4)	4 (7.7)	20 (38.5)	
	Bone	7 (13.5)	3 (5.8)	6 (11.5)	16 (30.8)	
	Liver	0 (0.0)	8 (15.4)	2 (3.8)	10 (19.2)	
	Lymph node	1 (1.9)	1 (1.9)	1 (1.9)	3 (5.8)	
	Adrenal gland	1 (1.9)	2 (3.8)	0 (0.0)	3 (5.8)	
Time from initial treatment to SBRT (month)						0.825
	Median (range)	25(2-118)	52 (11-130)	39 (17-145)	44 (2-145)	
Number of metastases, N (%)						0.353
	1	13 (28.9)	14 (31.1)	13 (28.9)	40 (88.9)	
	2 or 3	2 (4.4)	3 (6.7)	0 (0.0)	5 (11.1)	
Systemic therapy at the diagnosis of OMD, N (%)						0.153
	Yes	2 (4.4)	6 (13.3)	6 (13.3)	14 (31.1)	
	No	13 (28.9)	11 (24.4)	7 (15.6)	31 (68.9)	
Systemic therapy after SBRT, N (%)						0.009
	Yes	1 (2.2)	1 (2.2)	6 (13.3)	8 (17.8)	
	No	14 (31.1)	16 (35.6)	7 (15.6)	37 (82.2)	
Maximal tumor diameter (mm), N (%)						0.721
	≥20	9 (17.3)	12 (23.1)	9 (17.3)	30 (57.7)	
	<20	8 (15.4)	10 (19.2)	4 (7.7)	22 (42.3)	
BED (α/β=10), N (%)						0.041
	<80	9 (17.3)	4 (7.7)	7 (13.5)	20 (38.5)	
	≥80	8 (15.4)	18 (34.6)	6 (11.5)	32 (61.5)	

The numbers of patients who had de novo, repeat, and induced OMD were 15 (33.3%), 17 (37.8%), and 13 (28.9%), respectively. Eight patients (one de novo, one repeat, and six induced) continued systemic therapy after SBRT. Approximately half (46.2%) of the patients with induced OMD continued systemic therapy following local SBRT. In comparison, only a minority of patients in the de novo and repeat OMD groups (6.7% and 5.9%, respectively) received systemic therapy. Significant differences were detected in the sex and the utilization rates of systemic therapy (p=0.041 and p=0.009, respectively). The post hoc test revealed a trend of higher utilization of systemic therapy in the induced OMD group compared to the de novo OMD and repeat OMD groups (p=0.086 and p=0.074).

Significant difference was also observed in biologically effective dose (BED) using α/β=10 (BED10) between the three groups. The median BED10 of the prescribed dose was 80 Gy (range: 43.2-105 Gy). The most prescribed dose per fractionation was 42 Gy/four fractions (18 treatment sites, 34.6%). The pattern of the dose fractionation regimen used at each treatment site is shown in Table 2.

**Table 2:** Dose fractionation regimen used in each treatment site BED10: biologically effective dose using α/β=10

Physical dose fractionation (BED10)	Prescription isodose line (%)	Treatment site	Treatment site number (%)
42 Gy/four fractions (86.1 Gy)	80	Lung	18 (34.6)
40 Gy/four fractions (80 Gy)	70	Liver	8 (15.4)
35 Gy/five fractions (59.5 Gy)	70-80	Bone and liver	5 (9.6)
24 Gy/two fractions (52.8 Gy)	75-80	Bone	5 (9.6)
42 Gy/10 fractions (59.6 Gy)	80	Bone	3 (5.8)
45 Gy/five fractions (85.5 Gy)	80	Adrenal gland	2 (3.8)
40 Gy/five fractions (72 Gy)	80	Lymph node	2(3.8)
30 Gy/three fractions (60 Gy)	75-80	Bone	2 (3.8)
Others			7 (13.5)

During the follow-up period, local recurrence was observed in five patients: two with hepatocellular carcinoma and one with breast, bladder, and lung cancer. These patients had simultaneous or previous recurrence at other sites; therefore, no patients developed local recurrence only at the irradiated site. The local control rates at one year for the entire, de novo, repeat, and induced cohorts were 87.2%, 87.5%, 90.2%, and 83.9%, respectively (p=0.80). The one-year PFS rates in patients in the de novo, repeat, and induced OMD groups were 35.0%, 56.7%, and 29.9%, respectively (p=0.58). The one-year OS rates in patients in the de novo, repeat, and induced OMD groups were 80.0%, 86.2%, and 80.8%, respectively (p=0.50).

**Figure 2:**
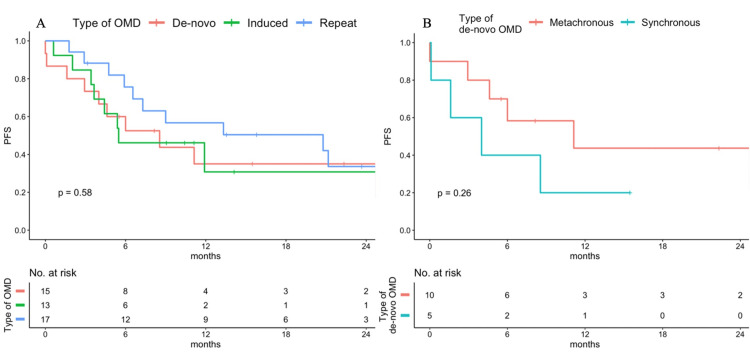
The progression-free survival (PFS) rate of the subgroups per the ESTRO/EORTC classification (A) and de novo OMD cohort stratified by chronicity (B) One patient with metachronous OMD developed distant metastasis during the course of the treatment, resulting in the survival curve starting below 1.0 ESTRO/EORTC, European Society for Radiotherapy and Oncology/European Organisation for Research and Treatment of Cancer; OMD, oligometastatic disease

Figure 2A shows the Kaplan-Meier analysis of PFS stratified by metastatic state. There were no significant differences in the PFS and OS among the three groups.

The median times to progression in the de novo, repeat, and induced OMD groups were eight, 21, and 10 months, respectively (p=0.58). Among patients in the de novo OMD group, one-year PFS in patients with synchronous and metachronous OMD was 20.0% and 43.8%, respectively (p=0.30) (Figure 2B). In the univariate analysis, PS and a BED10 lower than 80 Gy were significantly associated with shorter PFS. Regarding histology, both hepatocellular carcinoma and breast cancer were significantly associated with shorter PFS (Table 3). In the multivariate analysis, PS was the only significant predictor of PFS.

**Table 3:** Univariate and multivariate analysis of variables associated with PFS PFS, progression-free survival; HR, hazard ratio; PS, performance status; SBRT, stereotactic body radiation therapy; BED, biologically effective dose

		PFS HR (95% CI)	P-value	PFS HR (95% CI)	P-value
		Univariate		Multivariate	
Classification	De novo	Reference		Reference	
	Repeat	0.65 (0.27-1.58)	0.34	0.39 (0.11-1.42)	0.15
	Induced	0.97 (0.38-2.49)	0.96	0.62 (0.22-1.70)	0.35
PS	0-1	Reference		Reference	
	2	3.47 (1.32-9.15)	0.01	3.31 (1.18-9.26)	0.02
Systemic therapy after SBRT	No	Reference			
	Yes	1.87 (0.74-4.69)	0.18		
Number of metastases	1	Reference			
	>1	2.41 (0.89-6.56)	0.08		
BED (α/β=10)	≥80	Reference		Reference	
	<80	3.06 (1.41-6.65)	0.005	1.65 (0.56-4.84)	0.36
Tumor diameter (mm)	≥20	Reference			
	<20	0.49 (0.22-1.10)	0.08		
Primary site	Lung	Reference		Reference	
	Liver	2.91 (1.03-8.18)	0.04	3.52 (0.89-13.9)	0.07
	Bladder or ureter	0.55 (0.07-4.42)	0.57	1.02 (0.11-9.8)	0.98
	Breast	3.83 (1.01-14.49)	0.05	3.51 (0.64-19.4)	0.15
	Others	4.22 (1.56-11.42)	0.005	2.46 (0.68-8.90)	0.17
Time from initial treatment to SBRT (month)	≤48	Reference			
	>48	0.80 (0.38-1.70)	0.57		

There were no grade 4 or higher toxicities related to radiation therapy. Grade 2 or higher acute AEs included grade 2 inflammation of the middle ear (one patient) and grade 2 radiation pneumonitis (one patient). Late AEs occurred in three patients, including two with grade 2 radiation pneumonitis and one with grade 3 radiation pneumonitis. The incidence of grade 2 or higher AEs at one year of observation was 11.1%.

## Discussion

In this retrospective study, patients with repeat OMD had a trend of longer PFS than those with de novo and induced OMD. In the de novo OMD group, patients with metachronous OMD had a trend of longer PFS than patients with synchronous OMD.

Several clinical phase II trials have recently shown that SBRT prolongs PFS and OS in patients with OMD [2-4]. However, it remains unclear which subgroups of patients are suitable for local ablative therapy. Moreover, clinical trials for repeat and induced OMD are limited [8], and the efficacy of local treatment for these cohorts needs to be more apparent.

Guckenberger et al. propose that repeat OMD may represent a subgroup with low metastatic capacity. This suggestion is based on the fact that while patients with repeat OMD have experienced failure following systemic or local therapy, they have not developed polymetastatic disease, which is the most common pattern of disease progression [8]. We investigated the prognosis of OMD after local SBRT, stratified by the ESTRO/EORTC classification and multiple other factors. Although there was no significant difference in PFS or OS among the three groups of OMD, the PFS of patients in the repeat OMD group at one year was 56.7%, showing a trend toward longer disease control among the three groups. This may reflect the low metastatic capacity of repeat OMD, as described by Guckenberger et al. [8]. Klement et al. showed that appropriately selected patients (such as younger age and good local control) with a history of repeat SBRT may have safety and efficacy profiles comparable to those with single lung metastasis treated with SBRT [14]. Furthermore, a previous study suggested that a cure may be achieved in patients with repeat OMD by treating recurrent lesions with local therapy [15]. Although further investigation is needed, local therapy may be a good option for patients with repeat OMD.

In this study, patients with metachronous OMD had a trend of longer PFS than those with synchronous OMD, with one-year PFS of 43.8% and 20%, respectively. The main reason why metachronous OMD has a better prognosis than synchronous OMD is that metachronous OMD has slower growth than synchronous OMD and is diagnosed earlier [16]. Some studies [17,18] have shown that patients with metachronous OMD have prolonged OS than patients with synchronous OMD. Regarding PFS, Friedes et al. created a nomogram that predicts PFS in patients with oligometastatic lung cancer. Among the four factors (number of metastases, targetable mutation, intracranial metastases, and timing of metastases) that constitute this nomogram, synchronous OMD is the most influential prognostic factor for PFS [19].

Previous randomized phase II trials demonstrated a PFS rate of 35%-50% at one year in patients with OMD whose disease was otherwise controlled with systemic therapy [2-4]. In these trials, 55%-100% [3,4] of the patients continued systemic therapy following SBRT. In contrast, our study showed a favorable PFS in patients with repeat OMD, with a one-year PFS of 56.7%, despite the low rate of systemic therapy maintenance following SBRT. Patients with a long history of treatment, especially those with repeat OMD, often have limited options for systemic therapy. Although further studies are needed, local therapy may be an effective alternative to systemic therapy in these patients.

Previous reports showed that patients in the induced OMD group performed poorly in terms of PFS and OS than those in the other groups [17]; however, our study did not find any significant differences in PFS and OS between the induced OMD and other groups. In this study, approximately half of the patients with induced OMD continued systemic therapy after SBRT. In contrast, most patients with repeat OMD and de novo OMD did not continue systemic therapy after SBRT. Given the discordance of systemic therapy use between cohorts, we may be underestimating the survival benefit from local ablative therapy in patients with de novo and repeat OMD.

In our study, the group with a BED10 of 80 Gy or higher (equivalent total dose in 2 Gy fraction {EQD2} (α/β=10)≥66.7 Gy) exhibited longer PFS, compared to the group with a BED below 80 Gy (EQD2 (α/β=10)≤62.5). The threshold dose was slightly higher than previously reported by Ito et al., where colorectal or gynecological solitary oligometastatic lesions receiving 60 Gy (EQD2 (α/β=10)) or higher demonstrated favorable LC and OS [20]. Admittedly, our study included only a limited number of colorectal and gynecological cancer patients; however, an improved prognosis might be expected by delivering a higher BED to the metastatic lesions. Lower PS (2 versus 0-1) and histology indicating breast or hepatocellular cancer are also associated with shorter PFS, consistent with a previous study [21]. Chen et al. demonstrated that OMD patients with breast cancer showed shorter PFS [22]. In the context of OMD with hepatocellular carcinoma, local therapy for oligometastatic lesions improves both PFS and OS [23]. However, to the best of our knowledge, no studies have compared the prognosis of hepatocellular carcinoma with that of other histological types. The efficacy of radiation therapy for oligometastases in hepatocellular carcinoma remains to be elucidated in future research.

A recently published meta-analysis of SBRT for OMD estimated the incidence of grade 3-5 acute and late AEs to be less than 10% [24]. In the SABR-COMET trial protocol, dose fractionations for lung lesions were 54 Gy/three fractions (biologically effective dose using α/β=3 {BED3}=378 Gy), 55 Gy/11 fractions (BED3=256 Gy), and 60 Gy/eight fractions (BED3=210 Gy) [3,11], and two of the 34 patients treated for lung lesions experienced grade 5 AEs (pneumonitis and lung abscess at the irradiation site). On the other hand, we mainly employed 42 Gy/four fractions (BED3=189 Gy), resulting in only one grade 3 or higher acute AE (2.2%). Nevertheless, the local control rate at one year was approximately 90%, which was generally comparable to previous studies [25].

This study had several limitations. First, the median follow-up period was 14 months, which may not fully reflect the efficacy of the local treatment. However, Gomez et al. reported that the superiority of PFS at one year with the local treatment of OMD was maintained in a long-term study as well [26]. In a retrospective analysis of patients with OMD by Willmann et al., the PFS curves fell predominantly in the first 12 months, and the PFS rate was generally maintained after one year across all three groups according to the ESTRO/EORTC classification [17]. The PFS at one year in this study will likely be maintained over a long-term period. Second, the sample size of this study was small. The small proportion of patients with synchronous OMD makes comparison with previous studies difficult. In addition, patient and tumor characteristics were heterogeneously distributed among the three groups. The findings derived from current study may not be applied to the tumor types that are less prevalent in our study. Third, because this study was conducted at a single institution, there may be bias in the primary tumor, histology, and irradiation sites. Lastly, because this was a retrospective study, systemic therapy and radiation dose fractionation were not standardized for each patient. As the candidates for local SBRT were partly selected by referring physicians, the study may have included only some of the patient groups defined by the ESTRO/EORTC.

## Conclusions

Local SBRT was well tolerated in patients with OMD. BED10 of 80 Gy or higher was associated with longer PFS. Patients with repeat OMD showed a trend of favorable PFS, suggesting that this subgroup may benefit from local therapy at metastatic sites. Future prospective studies should validate this finding.
